# Steady-State Visual Evoked Potentials Can Be Explained by Temporal Superposition of Transient Event-Related Responses

**DOI:** 10.1371/journal.pone.0014543

**Published:** 2011-01-18

**Authors:** Almudena Capilla, Paula Pazo-Alvarez, Alvaro Darriba, Pablo Campo, Joachim Gross

**Affiliations:** 1 Centre for Cognitive Neuroimaging (CCNi), Institute for Neuroscience and Psychology, University of Glasgow, Glasgow, United Kingdom; 2 Department of Biological and Health Psychology, Autonoma University of Madrid, Madrid, Spain; 3 Department of Clinical Psychology and Psychobiology, University of Santiago de Compostela, Santiago de Compostela, Spain; 4 Center of Magnetoencephalography Dr Perez Modrego, Complutense University of Madrid, Madrid, Spain; 5 Laboratory of Cognitive and Computational Neuroscience, Center for Biomedical Technology, Complutense University of Madrid-Polytechnic University of Madrid, Madrid, Spain; 6 Department of Basic Psychology, Autonoma University of Madrid, Madrid, Spain; 7 School of Psychology, University of Glasgow, Glasgow, United Kingdom; Cuban Neuroscience Center, Cuba

## Abstract

**Background:**

One common criterion for classifying electrophysiological brain responses is based on the distinction between transient (i.e. event-related potentials, ERPs) and steady-state responses (SSRs). The generation of SSRs is usually attributed to the entrainment of a neural rhythm driven by the stimulus train. However, a more parsimonious account suggests that SSRs might result from the linear addition of the transient responses elicited by each stimulus. This study aimed to investigate this possibility.

**Methodology/Principal Findings:**

We recorded brain potentials elicited by a checkerboard stimulus reversing at different rates. We modeled SSRs by sequentially shifting and linearly adding rate-specific ERPs. Our results show a strong resemblance between recorded and synthetic SSRs, supporting the superposition hypothesis. Furthermore, we did not find evidence of entrainment of a neural oscillation at the stimulation frequency.

**Conclusions/Significance:**

This study provides evidence that visual SSRs can be explained as a superposition of transient ERPs. These findings have critical implications in our current understanding of brain oscillations. Contrary to the idea that neural networks can be tuned to a wide range of frequencies, our findings rather suggest that the oscillatory response of a given neural network is constrained within its natural frequency range.

## Introduction

It is a common practice to classify electrophysiological brain responses as either transient or steady-state [1]–[3]. On one hand, stimulation at low rates elicits transient event-related potentials (ERPs) characterized by a series of well known deflections [4]. On the other hand, rapid periodic stimulation produces a brain response characterized by a “quasi-sinusoidal” waveform whose frequency components are constant in amplitude and phase, the so-called steady-state response (SSR) [2], [5]. The traditional motivation for dividing the electrophysiological literature into the ERP and the SSR fields is summarized in the following statement: “*If the brain responded in a linear fashion, steady-state responses would be completely predictable from the transient response. However, the brain is not linear, and steady-state and transient responses therefore provide independent views of its function*” ([1] pg 178; see also [2], [6], [7]). From this point of view, SSRs are thought to be generated by the entrainment of a neural oscillation driven by the stimulation train [8]–[10]. However, an alternative hypothesis states that SSRs can be fully explained by the temporal superposition of transient ERPs [11]–[15]. A convincing demonstration that SSRs can be completely predicted from transient responses would indicate that both phenomena are probably generated by the same mechanism. It would thus lead to a more unified view of electrophysiological brain responses.

Surprisingly, despite its importance for interpreting experimental findings, the relation between SSRs and ERPs has received little attention. The exception is a series of studies that have explored the superposition hypothesis on auditory SSRs. In brief, some of these studies show that SSRs may be expressed as the temporal superposition of transient responses [15]–[17], whereas others fail to find such a simple relationship [18], [19]. This discrepancy in the results strongly depends on how the term “transient” response has been understood. Transient ERPs are traditionally defined as the response to an isolated or infrequent stimulus that provide enough time to the system to return to its initial state before onset of the next stimulus [1], [7]. Studies employing this concept of transient response have shown a poor reconstruction of SSRs from the superposition of transient ERPs, supporting the notion that ERPs and SSRs are non-linearly related. However, transient ERPs can also be understood as the transient response to a single event, either isolated or embedded in a stimulation train. Although these single-event transient responses are not directly accessible in steady-state stimulation sequences, they might be indirectly estimated from jittered stimulation sequences with a mean stimulation frequency close to the steady-state rate of interest. Studies using this approach have shown a linear relationship between rate-specific transient ERPs and SSRs, hence supporting the superposition hypothesis [15], [16].

This study aimed at investigating whether SSR generation can be explained by the temporal superposition of single-event transient ERPs without the need to appeal to oscillatory entrainment mechanisms. For this purpose we performed the following three tests that will be explained in more detail below. First, we directly tested the superposition hypothesis by comparing recorded and synthetic SSRs obtained from the linear addition of single-event transient responses. The second test aimed to rule out the possibility that rate-specific transient ERPs estimated from jittered sequences were influenced by oscillatory entrainment. Third, we evaluated the existence of brain activity outlasting the stimulation train as this would be a clear indication of oscillatory entrainment.

As outlined above, we first tested whether SSRs can be predicted by the temporal superposition of rate-specific transient ERPs. This question has been addressed by the previously mentioned studies, although these have been confined to auditory SSRs. Importantly, if the superposition hypothesis is correct, it should account for SSR generation independently of sensory modality. Therefore, we specifically designed a study to investigate the superposition hypothesis in the generation of visual SSRs. In two different experiments we recorded brain potentials evoked by a patterned stimulus reversing at regular (i.e. isochronic) and non-regular (i.e. jittered) intervals at a wide range of stimulation rates. We estimated templates of transient visual ERPs separately for each stimulation rate from the temporally jittered sequences. The transient templates were sequentially shifted and linearly added in order to recreate synthetic responses at each stimulation rate. In the first experiment we explored the superposition hypothesis in a single occipital lead for stimulation rates ranging from 2.7 to 20 reversals per second (rev/s). The aim of Experiment 2 was, first, to replicate the results from the previous experiment for a more extensive set of typical SSR frequencies (>7 rev/s); and, second, to investigate whether the superposition hypothesis explains, not only the generation of visual SSRs in a single posterior lead, but also the scalp topographies elicited by different stimulation frequencies. In brief, we hypothesized that if SSRs and ERPs were two qualitatively different phenomena, synthetic and recorded waveforms and topographies would show a low correspondence, particularly at higher steady-state stimulation rates. Conversely, if SSRs and ERPs were linearly related, synthetic waveforms and topographies would accurately predict the recorded ones at any stimulation rate.

However, an accurate reconstruction of SSRs based on transient responses does not necessarily rule out the involvement of oscillatory entrainment in SSR generation. In theory, it is possible that jittered stimulation close to steady-state rates entrained a neural rhythm fluctuating in instantaneous frequency. If this happened, the stimulus-locked average of jittered sequences would result in a time-limited oscillatory wave similar to a transient ERP; and the temporal superposition of this entrained waveform would also produce a good reconstruction of SSRs. We explored this possibility, which has not been addressed by previous studies, by comparing the features of the stimulus-locked average waveforms obtained from jittered sequences to the features that should be expected in both situations: (i) entrainment of an oscillation with fluctuating frequency, and (ii) superposition of a “fixed” transient response. Briefly, the shape of the average ERP produced by an entrainment mechanism is expected to change as a function of both global (i.e. mean) and local (i.e. immediate) stimulation frequency. In contrast, the shape of the average ERP obtained from a superposition mechanism should be stable independently of both global and local stimulation frequency.

The third test aimed to further investigate the role of oscillatory entrainment in SSR generation. Specifically, we evaluated the existence of additional activity outlasting the stimulus train, as it would be expected if a neural oscillation had been entrained. Previous studies have shown either the presence of additional cycles of activity [17], [19] in some individual subjects or, alternatively, no sign of activity beyond the stimulus train [20]. However, these observations are often of a qualitative nature and have not been statistically tested. In this study we quantified and statistically test the presence/absence of activity beyond the stimulation sequence by time-locked averaging the responses to the last stimulus of the train.

Our results demonstrate that superposition of transient responses can completely explain SSRs if transient responses are constructed to capture the non-linearities related to adaptation phenomena. Furthermore, we did not find evidence of entrainment of a neural oscillation at the stimulation frequency, as the shape of the transient ERPs obtained from jittered sequences was stable, and there was no evidence of additional activity outlasting the stimulus train. We will discuss the implications of our findings for understanding the mechanisms of oscillatory brain responses. Specifically, we will focus on how some phenomena typically related to SSRs, such as oscillatory entrainment and resonance, might be explained without the need to invoke additional non-linear mechanisms.

## Materials and Methods

### Participants

Twenty-four healthy subjects participated in the study. Twelve subjects (6 males; mean ± SD age, 19.2±1.3) participated in the first experiment, and 12 subjects (12 females; mean ± SD age, 18.5±0.8) took part in the second experiment. All participants were right handed and had normal or corrected-to-normal vision. Subjects neither had ever suffered an epileptic seizure nor had a family history of epilepsy. Informed written consent was obtained from all subjects before participation. The study was approved by the corresponding local ethics committees and conducted in conformity with the declaration of Helsinki.

### Stimuli and procedure

**Figure 1:**
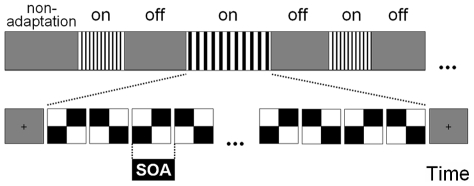
Stimulation design. ‘On’ and ‘off’ sequences were alternatively presented. ‘On’ sequences consisted of checkerboard reversal sweeps of 22 stimuli. The stimulus onset asynchrony (SOA) between pattern reversals was either constant (isochronic conditions) or variable (jitter conditions). The presentation of isochronic and jittered sequences at different rates was randomized. ‘Off’ sequences consisted of grey stimuli lasting 2 to 5 seconds. Note: checkerboards comprised 8 by 8 checks; the figure shows 2 by 2 checks for illustrative purposes.

In both experiments, participants seated in a comfortable chair at 1 m from a 43 cm monitor cathode ray tube (CRT), were requested to maintain their gaze at the centre of the screen where a fixation mark was placed during the ‘off’ periods (see Fig. 1). They were instructed to avoid horizontal eye movements or blinking during the presentation of stimulus sequences.

Stimuli consisted of checkerboards of size 8 by 8 checks with a pattern contrast of 96% and a mean luminance of 40 cd/m^2^. Each check subtended 1.4 degrees of visual angle. Spatially homogeneous grey stimuli displayed in pauses and around the pattern stimuli had the same average luminance as the checkerboards.

Stimuli were presented using the Presentation software (Neurobehavioral Systems, www.neurobs.com). Each run started with the presentation of a homogeneous grey stimulus for 15 sec to ensure a non-adaptational state. Twenty alternate ‘on’/‘off’ sequences were presented in each experimental run (Fig. 1). ‘On’ sequences consisted of step-wise checkerboard reversal sweeps of 22 stimuli. The sequence length was chosen to optimize the trade-off between the number of remaining stimuli after removing those affected by transient onset/offset effects and the influence of adaptation caused by longer sequence durations [21]. ‘Off’ sequences consisted of spatially homogeneous grey stimuli varying randomly from 2 to 5 seconds. Each subject participated in three runs.

Stimulation rate in ‘on’ sequences was defined as the number of reversals per second (rev/s). Given that a cycle is defined as a pair of pattern reversals (i.e. a patterned stimulus and its counter-phase reversal), a stimulation rate of 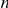 rev/s is equivalent to 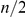 cycles/s or Hz. Different rates were generated by varying the stimulus onset asynchrony (SOAs). In order to avoid inaccuracies due to a constant monitor refresh rate, the exact frequency reversal rates were chosen in such a way that their corresponding SOAs were integer divisors of the monitor vertical refresh rate (100 Hz) [22]. Half of the ‘on’ sequences were isochronic (i.e. constant SOAs). The remaining ‘on’ sequences consisted of checkerboard reversal sweeps in jitter series with variable SOAs. The amount of jitter was randomly selected from a uniform distribution. This resulted in mean SOAs of each jitter condition that matched the SOA of its corresponding isochronic condition. In each run, stimulation rates and isochronic/jittered sequences were randomized, resulting in a total number of six stimulation sequences and 132 stimuli per condition.

In Experiment 1, patterned stimuli in the isochronic conditions were presented at the following rates: 2.7, 4.5, 7.1, 12.5 and 20 rev/s. This set of reversal frequencies comprises rates that typically elicit transient evoked responses (<3 rev/s) as well as rates characteristic of steady-state stimulation (>7 rev/s). The corresponding SOAs for the isochronic conditions were 370, 220, 140, 80 and 50 ms. The SOAs for the matched jittered conditions were 370±150, 220±80, 140±60, 80±30 and 50±20 ms (see Table 1).

**Table 1:** Experiment 1.

Isochronic conditions		Jitter conditions
Stimulation rate (rev/s)	SOA (ms)	SOA (ms)
2.7	370	370±150
4.5	220	220±80
7.1	140	140±60
12.5	80	80±30
20	50	50±20

Stimulation rates and corresponding SOAs for the stimulation conditions of Experiment 1. Abbreviations: SOA, stimulus onset asynchrony; ms, milliseconds; rev/s, reversals per second.

In Experiment 2, the isochronic conditions included one stimulation rate characteristic of transient ERPs (2.5 rev/s) and nine typical SSR reversal rates: 7.7, 8.3, 9.1, 10, 11.1, 12.5, 14.3, 16.7 and 20 rev/s. These stimulation frequencies corresponded to SOAs ranging from 130 to 50 in 10 ms steps. Unlike Experiment 1, the amount of jittering in the jitter conditions did not vary with stimulation rate, but was set constant to ±40 ms (see Table 2).

**Table 2:** Experiment 2.

Isochronic conditions		Jitter conditions
Stimulation rate (rev/s)	SOA (ms)	SOA (ms)
2.5	400	400±40
7.7	130	130±40
8.3	120	120±40
9.1	110	110±40
10	100	100±40
11.1	90	90±40
12.5	80	80±40
14.3	70	70±40
16.7	60	60±40
20	50	50±40

Stimulation rates and corresponding SOAs for the stimulation conditions of Experiment 2. Abbreviations: SOA, stimulus onset asynchrony; ms, milliseconds; rev/s, reversals per second.

### Recording of the EEG signal

In the first experiment, EEG activity was recorded with a NeuroScan system connected to a Synamps amplifier using an Ag/AgCl sintered electrode placed at the occipital pole (Oz according to the 10–20 international system) referred to the nose-tip and grounded with an electrode at FPz. Eye movements were monitored with electro-oculogram (EOG) electrodes attached above and at the lateral corner of the right eye. Electrode impedances were kept below 5 kΩ. EEG was continuously recorded at a sampling rate of 1000 Hz and filtered on-line with an analog bandpass of 0.05–100 Hz and a notch filter at 50 Hz.

In Experiment 2, the EEG signal was collected with BrainAmp amplifiers (BrainProducts, Munich, Germany) from 60 scalp sites using sintered Ag/AgCl electrodes mounted on an elastic cap (EASYCAP, Herrsching-Breitbrunn, Germany). EEG electrodes were placed following the extended 10–20 position system (Fp1, Fp2, AF7, AF3, AFz, AF4, AF8, F7, F5, F3, F1, Fz, F2, F4, F6, F8, FT7, FC5, FC3, FC1, FCz, FC2, FC4, FC6, FT8, T7, C5, C3, C1, Cz, C2, C4, C6, T8, TP7, CP5, CP3, CP1, CPz, CP2, CP4, CP6, TP8, P7, P5, P3, P1, Pz, P2, P4, P6, P8, PO7, PO3, POz, PO4, PO8, O1, O2, Oz) and referred to the tip of the nose. Four additional electrodes were placed above and below the left eye and on the outer canthi of both eyes to monitor blinks and eye movements. A single ground electrode was attached at nasion. As for Experiment 1, impedances were kept below 5 kΩ. The EEG signal was recorded unfiltered at a rate of 1000 Hz.

### Analysis of the EEG signal

**Figure 2:**
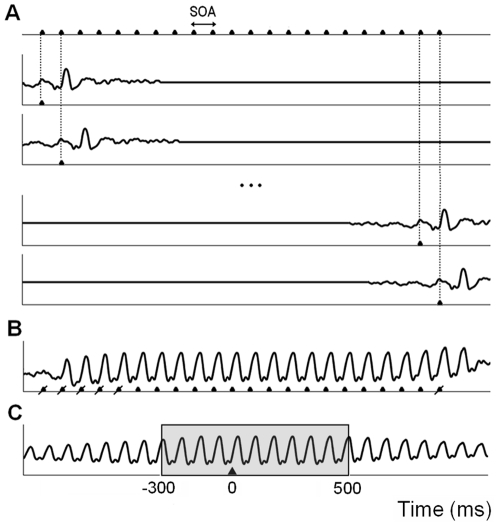
Procedure to synthesize steady-state responses. Synthetic data for each subject and stimulation rate were obtained by linear addition of the corresponding transient response template. **A**, The template was time-shifted at its corresponding SOA to create 22 synthetic trials. Here, we show as an example the transient template for the 12.5 rev/s condition of Experiment 1, time-shifted every 80 ms. **B**, The first five trials and the last one were discarded as for the recorded data. The remaining 16 synthetic trials were linearly added to simulate the response to one stimulation sequence. The triangles indicate stimulus onset. **C**, The synthetic signal was segmented into stimulus-locked epochs that were averaged. The epoch lasting from 300 ms pre-stimulus to 500 ms post-stimulus was extracted to be compared with its corresponding recorded waveform.

In both experiments, analysis of the data was performed using Matlab 7.5 (The MathWorks) and Fieldtrip (www.ru.nl/neuroimaging/fieldtrip/, 20091008 release). The EEG signal was digitally band-pass filtered between 1 and 60 Hz. Additionally, the 50 Hz electrical noise was removed in Experiment 2 by means of the discrete Fourier transform. Subsequently, the continuous EEG signal was segmented into epochs beginning at the onset of each patterned stimulus. The first five stimuli and the last stimulus of each sequence were not included in the analysis to eliminate pattern-onset/offset effects. This yielded a maximum number of 96 trials per condition (six stimulation sequences with 16 stimuli per sequence). Epochs covered a pre and post-stimulus interval that lasted the specific duration of the stimulation sequence for each condition (e.g. for the 2.7 rev/s condition the duration of the sequence was 5920 ms–370 ms by 16 stimuli – hence epochs lasted from 2960 ms before the stimulus to 2960 ms after the stimulus). Specifically, the first epoch was centered on the first stimulus of the first sequence; the second epoch was centered on the second stimulus of the first sequence, etc. EEG data were visually inspected for artifacts and contaminated sequences were rejected from additional analysis. The minimum number of trials for any condition after artifact rejection was 80. The generated stimulus-locked epochs were separately averaged and detrended for each condition. This averaging method provides a higher signal-to-noise ratio than the traditional averaging of entire stimulation sequences, since the number of epochs included in the average is considerably higher (e.g. 16 times higher in this case). It is important to note that the waveforms obtained through this averaging procedure show a decrease in amplitude as the distance from the zero time point increases (see Fig. 2), given that the number of brain responses included in the average for preceding/forthcoming stimulus positions is gradually reduced. After averaging, the mean across subjects (i.e. grand-average) waveforms were obtained for each isochronic condition and subjected to spectral analysis. Prior to spectral analysis time series were zero-padded (i.e. the signal was extended with zeros) up to a length of 6 s in Experiment 1, and 6.5 s in the Experiment 2, and windowed with a Hanning taper to compute the power spectra.

In Experiment 2, the scalp topographies at different phase angles of the dominant frequency at each isochronic condition were additionally computed. First, we calculated the complex frequency spectrum per condition. To obtain an appropriate spectral resolution for each dominant frequency, the spectra were computed over segments of data lasting 10 times the specific SOA of each condition (e.g. for the 7.7 rev/s condition, the data segment lasted 1300 ms, providing a spectral resolution of 0.77 Hz). Then, the temporal evolution of the amplitude (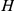) for a given frequency (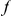) and phase angle (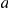) was computed for each electrode by combining the real (Re) and imaginary (Im) coefficients of the frequency spectrum as expressed in 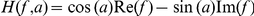 [23]. In sum, we extracted the amplitude for all electrodes at each dominant frequency from 0 to 180° phase angles in 45° steps, resulting in 5 topographic distributions per condition. Phase angles were referred to stimulus onset. Additionally, and in order to make topographies more comparable between conditions, we computed the topographies from 0 to 180° taking as temporal reference the common positive component that peaked at 100 ms.

### Test 1. Synthetic waveforms and topographies generated by superposition of transient ERPs

Synthetic SSRs generated from linear superposition of transient visual ERPs were modeled as follows (Fig. 2). First, templates of transient ERPs were estimated for each subject and each stimulation rate from the stimulus-locked averages of the corresponding jitter condition. For example, to create the transient template for the 2.7 rev/s condition the average for the jitter 2.7 rev/s condition was used. In Experiment 2, the transient templates were created by multiplying the ERPs obtained from the jittered conditions by a Gaussian function with a standard deviation of 50 ms and centered at 100 ms. This adjustment was necessary to minimize the influence of the ERPs elicited by preceding and forthcoming stimuli. Each template comprised the 300 ms pre-stimulus and 500 ms post-stimulus interval of the average waveform. This segment was zero-padded up to the total duration of the stimulation sequence of each condition. Then, the template was time-shifted at its corresponding SOA 22 times. After discarding the first 5 trials and the last one of each sequence, the remaining 16 synthetic trials were added to simulate the response to one stimulation sequence. Subsequently, the synthetic signal was subjected to the same analysis procedure described above for the recorded data; that is, the synthetic data were segmented into stimulus-locked epochs that were averaged and detrended for each condition. Then, grand-average waveforms and their related power spectral densities were obtained. In addition, the scalp topographies of the synthetic SSRs were computed in Experiment 2.

To statistically test the null hypothesis that recorded and synthetic waveforms and topographies were different we employed a percentile bootstrap approach [24]. The procedure, explained in detail below, was identical for testing differences between waveforms in both experiments and scalp topographies in Experiment 2. The statistical analysis of SSR waveforms was performed across the temporal dimension for each stimulation condition separately. The comparison between recorded and synthetic topographies was performed across electrode sites independently for both stimulation frequency and phase angle.

The procedure to test the differences between recorded and synthetic waveforms consisted of the following steps that were repeated 1000 times (see [25] for further details). First, pairs of recorded and synthetic waveforms were sampled with replacement across subjects. Then, we computed the 20% trimmed mean [24], [26] across subjects separately for the bootstrapped recorded and synthetic waveforms and subtracted them. In order to correct for multiple comparisons, we stored the maximum absolute value across time in each repetition. From the resulting distribution of bootstrapped estimates of the difference between recorded and synthetic waveforms, we computed the 95% confidence interval. Using this bootstrap technique, differences between waveforms are considered significant if the confidence interval does not include zero. As our working hypothesis was that recorded and synthetic waveforms do not differ, we also calculated the confidence interval using the least restrictive statistical criterion (i.e. p<.05, no multiple comparisons correction). This second test aimed to verify that potential differences between the waveforms were not masked by the use of a more conservative criterion for significance. The procedure for calculating the non-corrected 95% confidence interval was the same as explained above, with exception of the computation of the maximum absolute value across time of the difference between waveforms. Instead, the difference between the bootstrapped waveforms at each time point was stored in each repetition, leading to a specific distribution of bootstrapped estimates and a subsequent 95% confidence interval per time point.

In order to test whether recorded and synthetic topographies differed, we repeated the above procedure, computing in this case the difference between the bootstrapped topographies across electrodes, which led to a distribution of bootstrapped estimates. As for the analysis of SSR waveforms, we computed the corrected as well as the non-corrected 95% confidence intervals at each electrode.

### Test 2. Entrainment Vs. Superposition mechanisms in jittered sequences

This second test investigated the possibility that the templates of transient responses previously employed in the synthesis of SSRs might have been influenced by oscillatory entrainment. To this end, we first simulated average waveforms obtained from jittered sequences at three different stimulation rates (average frequency: 8,12 and 16 Hz; jitter: ±40 ms) in the following two situations. First, in the entrainment situation we simulated an oscillatory response with fluctuating instantaneous frequency based on the immediately past SOA. In the second situation, simulating the superposition mechanism, we convolved the same jittered stimulation sequence with a transient response, consisting of a Gaussian-weighted (standard deviation: 40 ms; centered at 0 ms) 12 Hz wave. We then evaluated how the shape of the stimulus-locked average response is influenced by both global (i.e. mean) and local (i.e. instantaneous) stimulation frequency. The effect of global stimulation frequency was simulated by computing the average response for each stimulus rate (8, 12 and 16 Hz) separately. The effect of local stimulation frequency was examined by grouping the trials of each condition by their immediately preceding SOA. Subsequently, the average response was computed for each group of trials separately. The short SOA group included those trials whose SOA was lower than the 33^rd^ percentile. Similarly, the medium SOA group comprised trials with SOA higher than the 33^rd^ percentile and lower than the 66^th^ percentile; and the long SOA group was defined by SOAs higher than the 66^th^ percentile. Additionally, and in order to facilitate the comparison between simulated and recorded data, we repeated this procedure for the same steady-state stimulation frequencies employed in Experiment 2 (ranging from 7.7 to 20 rev/s). We quantified the shape of the obtained average waveform by computing the peak-to-peak latency for all the global by local frequency combinations.

The above procedure was applied to the recorded jitter conditions from Experiment 2, as this provided a more extensive number of typical steady-state stimulation rates. In order to assess the effects of both global and local stimulation frequency on the shape of the stimulus-locked average responses, we split the trials with same global stimulation frequency in three groups based on their past SOA. We then computed the single-subject average waveforms for each global by local frequency combination, and quantified its shape by measuring the peak-to-peak latency. The negative and positive peaks, corresponding to N75 and P100 components respectively, were identified by means of an automatic procedure detecting local minima/maxima (taking into account ±2 neighbouring time points). The search for local minima was restricted to the 20–90 ms time window; whereas the search for local maxima was performed throughout the 85–130 ms time window. Those cases where no local maxima/minima were identified were treated as missed values. Also, in some particular cases where more than one time point fulfilled the criteria for local maxima/minima, the one closer to the expected latency of the corresponding component was selected. Finally the effects of both global and local stimulation frequency on the shape (i.e. peak-to-peak latency) of the average ERP were statistically assessed by means of a 2-way ANOVA across subjects.

### Test 3. Additional activity beyond the last stimulus of the train

The third test aimed to investigate the existence of oscillatory entrainment as reflected by additional activity beyond the end of the stimulation sequence. This test was performed for the typical steady-state stimulation rates of both isochronic and jitter conditions from Experiment 2. For each condition and subject, we computed the time-locked average with respect to the last stimulus of the sequence. The response to the last stimulus was quantified as the mean amplitude in the 85 to 115 ms time window, corresponding to the P100 component. Similarly the response beyond the last stimulus of the train was defined as the mean amplitude in the time window where an additional P100 component might be expected (i.e. 85–115 ms plus the specific SOA for each condition). We then employed one-side one-sample t-tests, first, to verify the presence of a time-locked response to the last stimulus of the train and, second, to statistically test the presence/absence of an additional response beyond the end of the sequence.

## Results

### Experiment 1

#### Recorded waveforms

**Figure 3:**
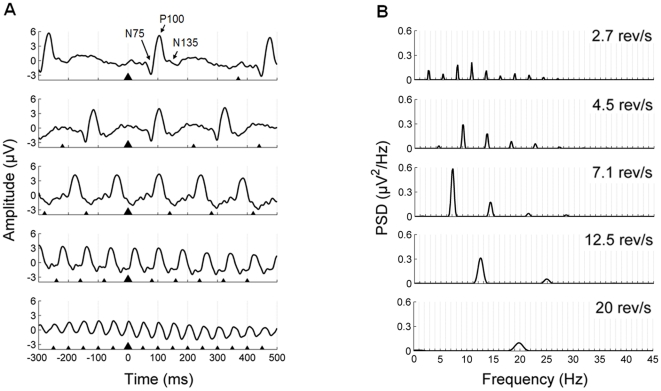
Recorded isochronic waveforms (Experiment 1). **A**, Grand-average waveforms (800 ms time window, including 300 ms pre-stimulus activity) for the five isochronic conditions. The typical deflections of the pattern reversal ERP (N75 at 70–90 ms, P100 at 80–120 ms and N135 at 120–180 ms) are indicated in the grand-average for the 2.7 rev/s condition. The triangles placed below the waveforms indicate the timing of stimuli appearance; the largest triangle represents the zero time point. **B**, Power spectral densities of the grand-average waveforms for the five isochronic conditions.

The grand-average waveforms and power spectral densities for the five isochronic conditions are shown in Figure 3. The large triangle represents the zero time point and the smaller triangles mark the onset of preceding and forthcoming stimuli. Although the typical deflections of the pattern reversal ERP (i.e. N75, P100 and N135; [4], [27]) become less identifiable as the stimulation rate increases, a positive deflection peaking at around 100 ms can be observed in all cases. As expected, the power spectra show prominent peaks at the fundamental frequency of stimulation and its harmonics.

**Figure 4:**
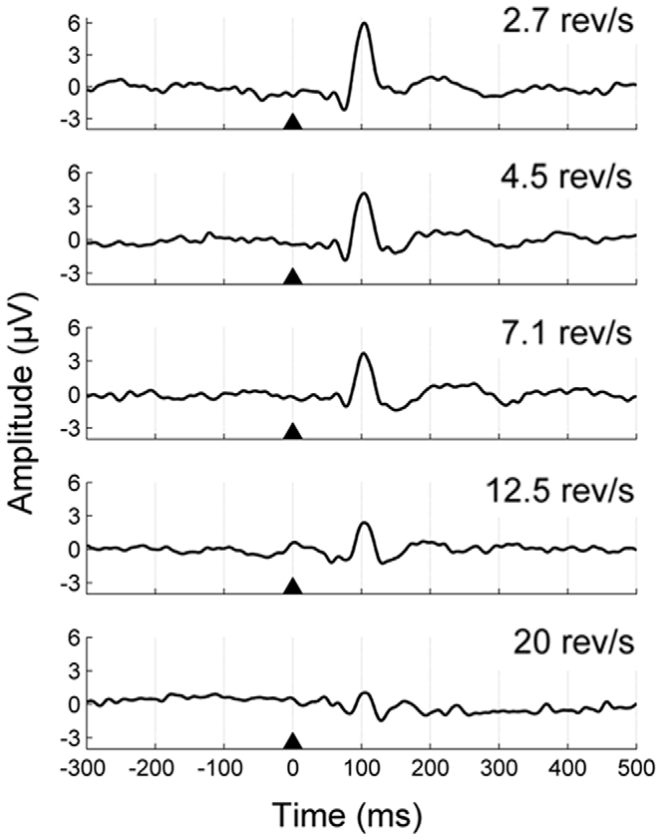
Templates of transient responses (Experiment 1). Grand-average waveforms (800 ms time window; 300 ms pre-stimulus) of the transient templates used to create the synthetic data at the five different rates. The transient templates correspond to the stimulus-locked average of the jitter conditions.

The grand-average waveforms for the jitter conditions that were employed as transient templates in the synthesis procedure are shown in Figure 4.

#### Synthetic waveforms

**Figure 5:**
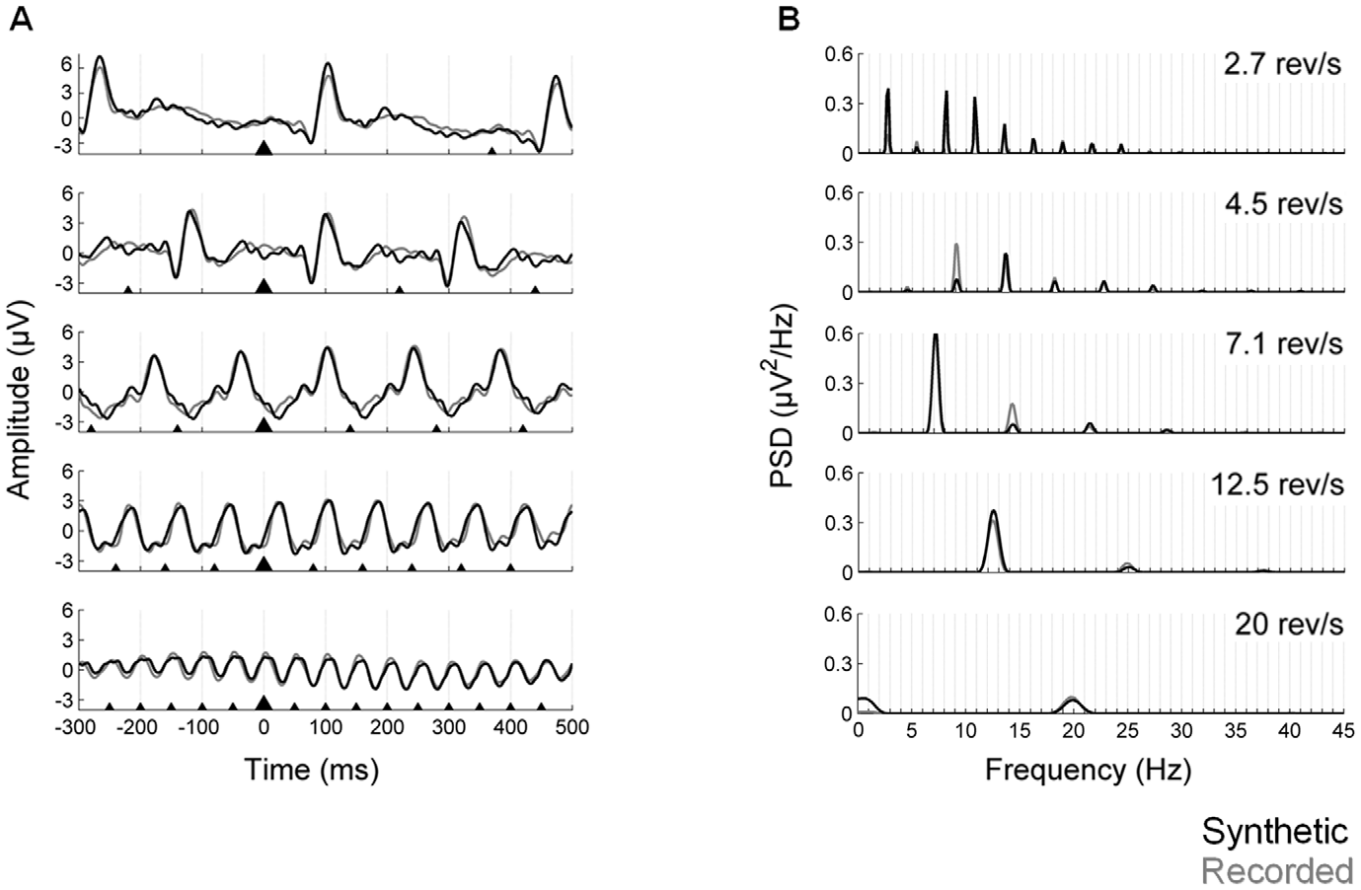
Synthetic waveforms obtained from linear superposition of transient templates (Experiment 1). The synthetic data are shown in black; the recorded data have been overlaid in grey for comparison. **A**, Grand-average waveforms (800 ms time window; 300 ms pre-stimulus) of the synthetic data at the five different rates. The triangles indicate stimuli onset; the largest triangle represents the zero time point. **B**, Power spectral densities of the grand-average waveforms.

The grand-averages and power spectral densities of the synthetic data at the five different rates are shown in Figure 5. The recorded and synthetic grand-average waveforms and power spectral densities have been overlaid to facilitate their comparison. As it can be observed, recorded and synthetic waveforms show a high correspondence in their amplitude–time pattern. Furthermore, power spectra of the synthetic data are characterized by pronounced peaks at the stimulation frequency and its harmonics, resembling the power spectra of the recorded data.

The supplemental material (Fig. S1) shows the recorded and synthetic data for two representative subjects at two different stimulation rates. The figure illustrates at a qualitative level that the synthetic data capture the rather large inter-individual differences of the recorded waveforms.

Finally, the results of the percentile bootstrap analyses revealed no significant differences between recorded and synthetic waveforms (p<.05, corrected for multiple comparisons) at any of the stimulation rates studied, as the 95% confidence intervals did contain zero in all cases. The confidence intervals for the five conditions, from lower to higher pattern reversal rate, were [−4.21, 2.98], [−3.17, 3.12], [−3.19, 3.66], [−3.21, 3.36] and [−2.99, 2.98]. When using a very liberal criterion for significance (i.e. p<.05, uncorrected) cyclic differences at a few time points emerged at the two lowest stimulation rates, 2.7 and 4.5 rev/s. These uncorrected significant differences represented only 1.5% of all the comparisons performed between recorded and synthetic waveforms.

### Experiment 2

#### Recorded waveforms

**Figure 6:**
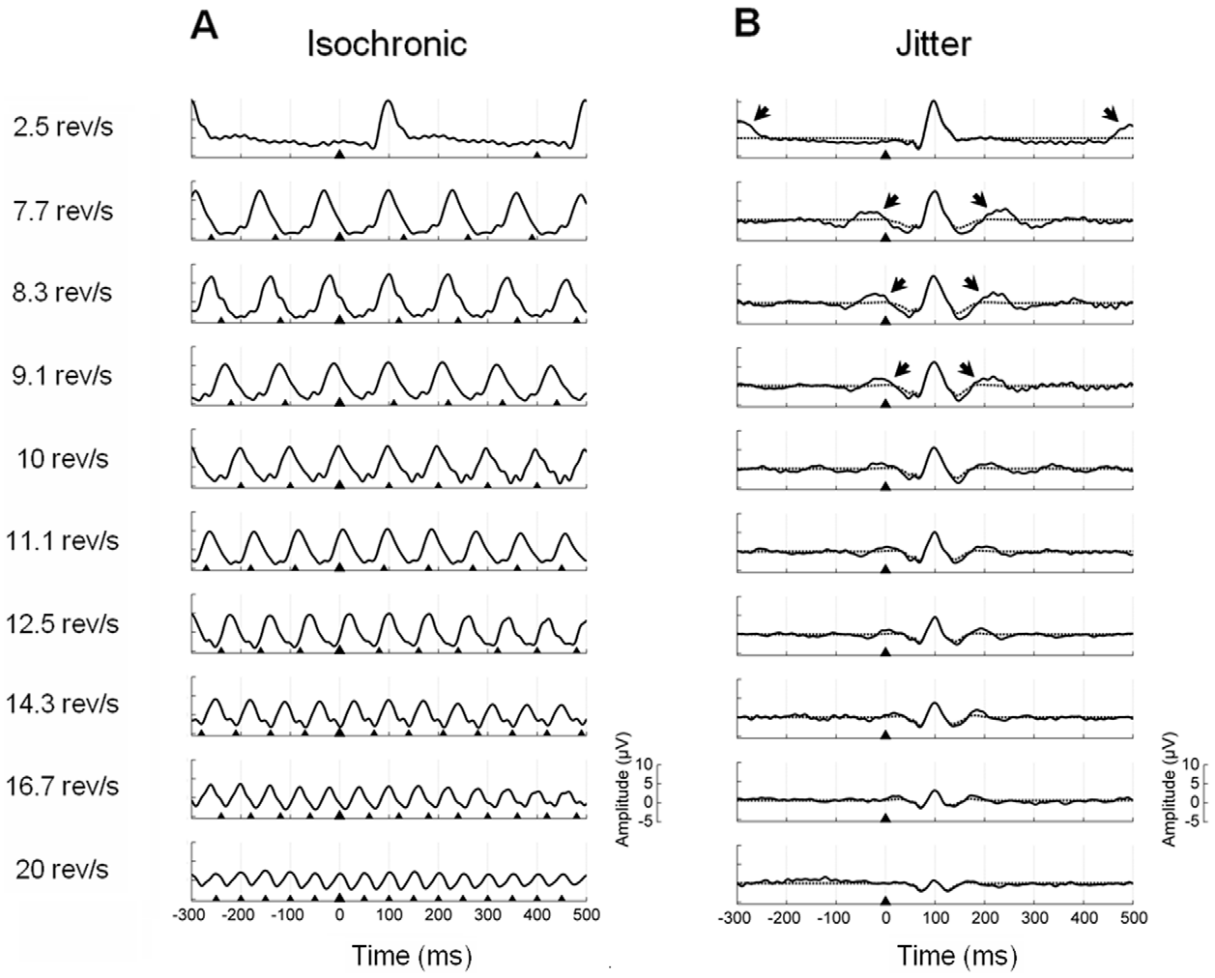
Grand-average waveforms at the Oz lead for both isochronic and jitter conditions (Experiment 2). **A**, Recorded waveforms (800 ms time window; 300 ms pre-stimulus) for the isochronic stimulation conditions, including one stimulation rate characteristic of transient ERPs (2.5 rev/s) and nine typical SSR reversal rates ranging from 7.7 to 20 rev/s. The triangles indicate stimuli onset; the largest triangle represents the zero time point. **B**, Grand-average waveforms of the corresponding jitter conditions are shown in solid line. Reminiscent activity from preceding and forthcoming stimuli has been marked by arrows. The Gaussian-modulated waveforms used as transient templates in the synthesis procedure are shown in dashed line.

The grand-average waveforms at the Oz lead for both isochronic and jitter conditions are shown in Figure 6. Unlike in the first experiment, the waveforms obtained from the jitter conditions exhibit some degree of time-locked activity related to preceding and forthcoming stimuli, as it has been indicated by arrows. This effect is more pronounced at lower stimulation rates and most likely due to the lower amount of jitter employed at these rates in this experiment. The Gaussian modulated waveforms that were used as transient templates in the synthesis procedure provided a more adequate estimation of the transient ERP by eliminating neighbouring responses, as can be observed in Figure 6.

#### Synthetic waveforms

**Figure 7:**
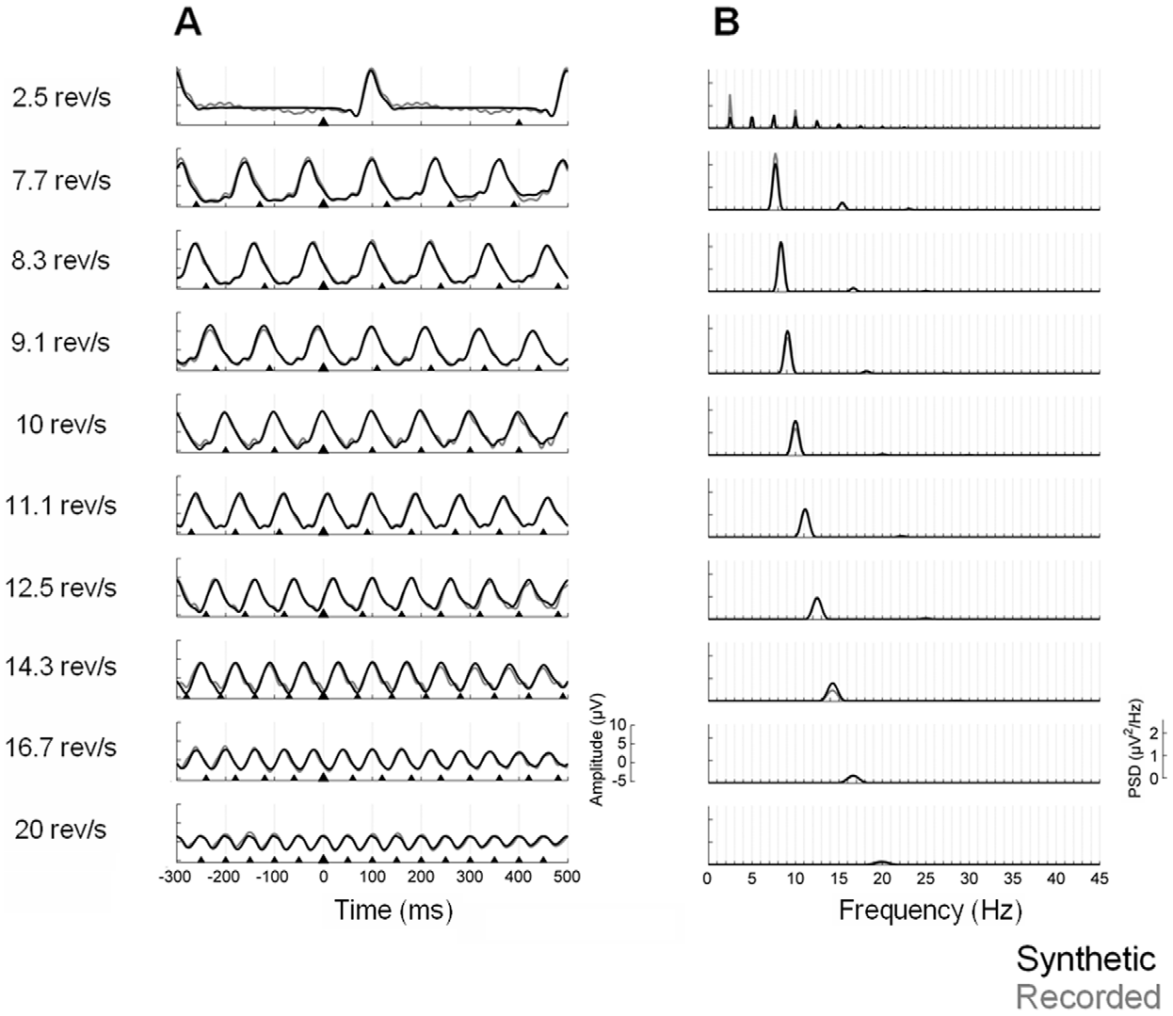
Synthetic waveforms obtained from linear superposition of transient templates (Experiment 2). The synthetic waveforms for the Oz lead at each stimulation rate are shown in black; the recorded data have been overlaid in grey for comparison. **A**, Grand-average waveforms of the synthetic data at the ten different rates studied. The triangles indicate stimuli onset; the largest triangle represents the zero time point. **B**, Power spectral densities of the grand-average waveforms.

The grand-average waveforms and power spectral densities of the synthetic data at Oz can be observed in Figure 7. As for the first experiment, the figure shows that synthetic SSR waveforms constructed by temporally adding transient ERPs highly resemble recorded SSRs. In addition, the power spectrum of synthetic waveforms also exhibited the characteristic spectrum of the pattern reversal SSR, namely dominant frequency responses at the fundamental frequency of stimulation when defined in rev/s or, equivalently, at the first harmonic (i.e. 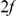) of the stimulation frequency when expressed in Hz.

The results of the statistical analyses quantitatively demonstrated the lack of differences between recorded and synthetic waveforms at all the stimulation rates studied (p<.05, corrected for multiple comparisons). The 95% confidence intervals, which did contain zero in all cases, were the following (from lower to higher stimulation rate): [−3.48, 4.34], [−3.35, 3.92], [−2.77, 3.12], [−3.76, 2.04], [−3.40, 3.04], [−2.68, 2.63], [−3.42, 2.95], [−3.13, 3.25], [−2.94, 2.71] and [−2.11, 3.16]. The results of the bootstrap analyses without multiple comparisons correction showed significant differences in 7.5% of all the possible comparisons between recorded and synthetic waveforms. This small decrease in the robustness of the statistical results with respect to the first experiment, where only 1.5% of all comparisons showed an uncorrected p-value below .05, is likely due to the less optimal procedure employed to estimate the transient templates in Experiment 2.

#### Recorded and synthetic topographies

**Figure 8:**
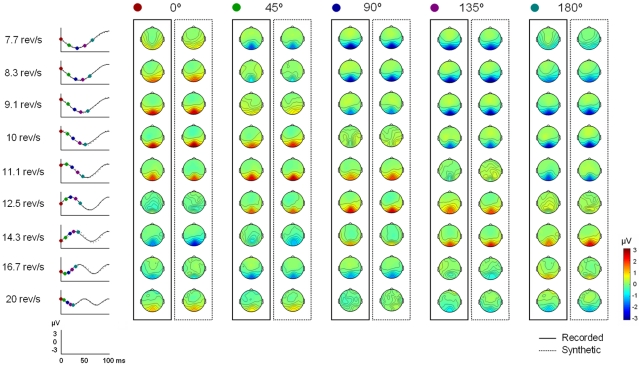
Voltage topography for both recorded and synthetic data (Experiment 2). Scalp voltage topographies for the dominant frequency of each condition (rows) and phase angle (columns) from 0° to 180°. Recorded topographies are presented in the solid boxes; synthetic topographies, in the dashed boxes. The time points corresponding to each phase angle are indicated by its corresponding colour in the time courses of the frequency amplitude at Oz shown on the left side. Phase angles are referred to stimulus onset (i.e. 0° phase angle corresponds to 0 ms).

The scalp topographies for both recorded and synthetic data are shown in Figure 8. The figure shows the topographic distribution of each dominant frequency amplitude at 5 different phase angles evenly spaced from 0 to 180°. The time course of the frequency amplitude at Oz during the first 100 ms after stimulus onset is shown on the left side of the figure to indicate the time points that correspond to each phase angle.

The voltage field showed a unitary focus over medial occipital scalp that shifted polarity across different phase angles and stimulation frequencies. Statistical analyses confirmed that although topographies varied across different frequencies they did not differ between recorded and simulated data (p<.05, corrected for multiple comparisons). At an uncorrected p<.05 significance level, only 8 electrode-frequency-angle combinations were significantly different for recorded and synthetic data (in the 7.7 rev/s condition at 0° and 180° in Pz, P2 and PO4, and at 135° in O1, Oz), representing 0.3% of all the comparisons performed.

The variation in scalp topography for different stimulation frequencies at equivalent phase angles seems to reflect the lack of correspondence between phases at stimulus onset for the different conditions. For example, as it can be observed in Figure 8, the time courses for the 9.1 and 14.3 rev/s conditions are in anti-phase at stimulus onset, resulting in topographies that are inverted in polarity. In order to obtain topographies referred to a common initial phase for all conditions, we additionally computed the temporal evolution of each frequency amplitude setting the initial 0° phase angle to the positive peak corresponding to P100. In this case, the time courses of each frequency amplitude at Oz were in cosine phase for all stimulation rates, and both recorded and synthetic topographies were characterized by a medial occipital positive focus at 0° that reversed in polarity at 180° (Fig. S2). In addition, the amplitude of the medial occipital focus showed lower intensity at higher stimulation rates, in agreement with the decrease in amplitude observed in the average waveforms (see Fig. 7). As for the scalp topographies referred to stimulus onset, bootstrap analyses of the topographies referred to P100 revealed no significant differences between recorded and synthetic topographies at a corrected p<.05 significance level. At the uncorrected p<.05 level only one combination, corresponding to the 0.04% of all possible comparisons, reached significance (TP8 in the 8.3 rev/s condition at 0°).

#### Effect of global and local stimulation frequency on the average ERP

**Figure 9:**
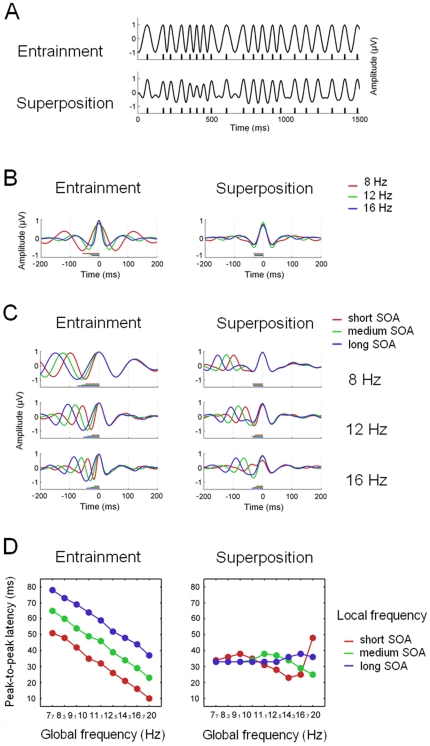
Simulation of the influence of both global and local stimulation frequency on the shape of the average ERP. **A**, Example of the response elicited by a jittered stimulation train in both the oscillatory entrainment and the superposition scenarios. **B**, Average waveforms for different stimulation frequencies. The solid bars along the x axis reflect the peak-to-peak latency. **C**, Average waveforms for trials grouped by their immediately past SOA. **D**, Variation in the peak-to-peak latency in both scenarios, as a function of both mean stimulation rate and local frequency.

The simulation of the effects of both global (i.e. mean) and local (i.e. immediate) stimulation frequency on the shape of the average transient response are shown in Figure 9. Figure 9A exemplifies the response elicited by a jittered stimulation sequence under the two studied situations, oscillatory entrainment and superposition of a “fixed” transient response. The stimulus-locked average waveforms for different stimulation frequencies under both scenarios are shown in Figure 9B. In the same vein, the average waveforms for trials with similar preceding SOA are shown in Figure 9C. Finally, the influence of both mean stimulation frequency and past SOA on the shape of the average response is summarized in Figure 9D. As it can be observed, in the oscillatory entrainment situation the peak-to-peak latency of the average waveform is influenced by both global and local stimulation frequency. More specifically, the higher the global stimulation rate the shorter the wave length and, similarly, the higher the preceding stimulation frequency (i.e. shorter SOAs) the shorter the peak-to-peak latency. To sum up, in the oscillatory entrainment case the shape of the obtained transient response changes accordingly to the frequency of stimulation. However, in the superposition case the shape of the average waveform is approximately stable, and it is not influenced by either global or local stimulation rate.

**Figure 10:**
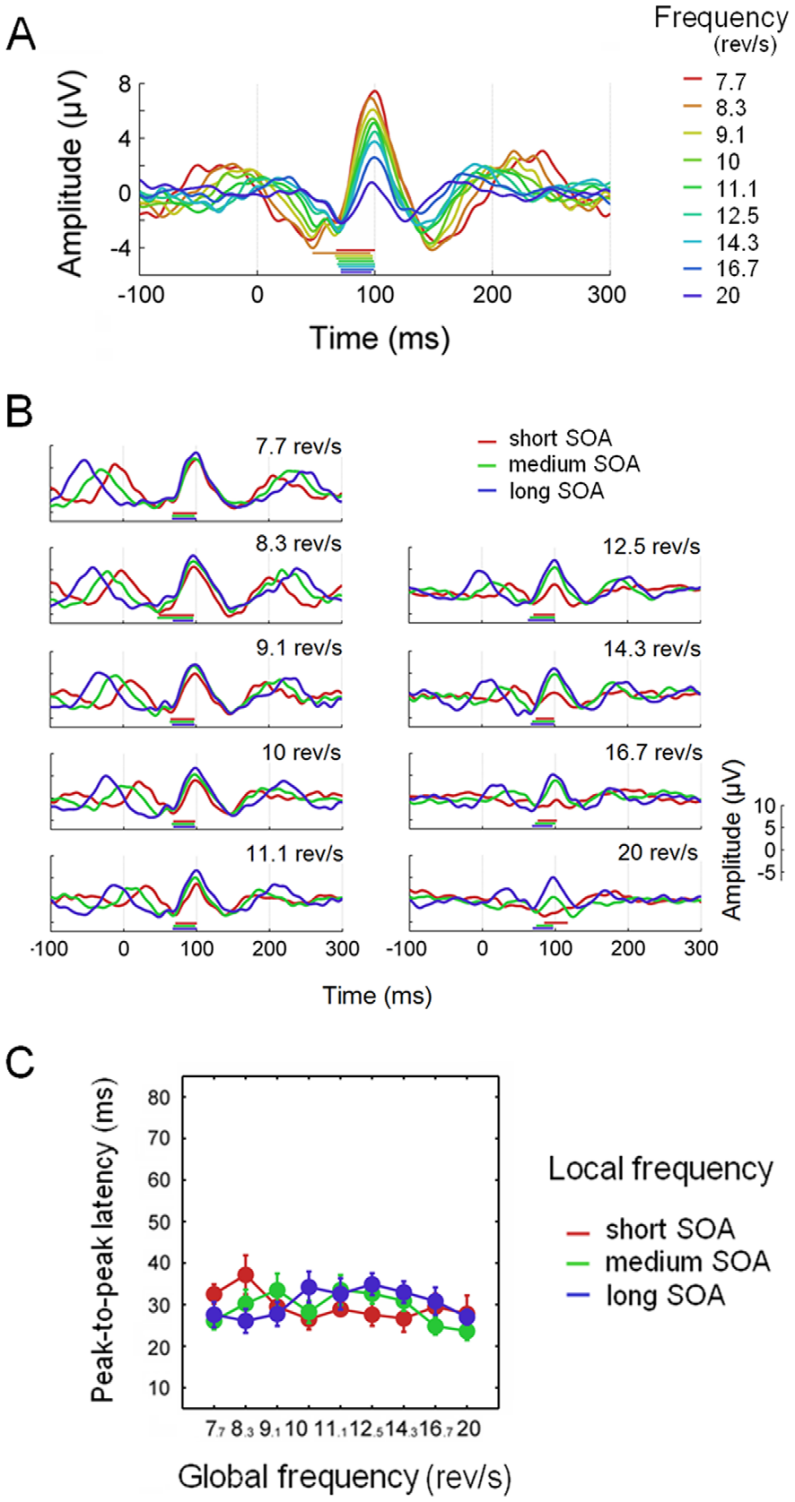
Effect of global and local stimulation frequency on the shape of recorded ERPs. **A**, Grand-average ERPs for the jitter conditions of Experiment 2. The solid bars along the horizontal axed indicate the peak-to-peak (N75-P100) latency. **B**, Grand-average ERPs for trials grouped by their immediately past SOA at each stimulation rate. **C**, Peak-to-peak latencies as a function of both global and local stimulation frequency. The error bars indicate the standard error of the mean across subjects.

The grand-average transient ERPs obtained from the jitter conditions of Experiment 2 are shown in Figure 10A. In order to facilitate the comparison of the waveforms at different stimulation frequencies, they have been superimposed, and the corresponding peak-to-peak latencies have been indicated along the x axis. The grand-average transient ERPs for the set of trials with similar past local frequency is shown in Figure 10B. As for the simulations, the variations in peak-to-peak latency as a function of both global and local stimulation frequency are shown in Figure 10C. Although the results are less clear for high stimulation frequencies (16.7 and 20 rev/s) and short SOAs, this is most likely due to a higher overlapping of waveforms due to the particularly short SOAs employed in these conditions (ranging approximately from 10 to 46 ms). As a matter of fact, the effect of higher overlapping as a consequence of extremely short SOAs can be already observed in the results of the simulations. In the simulation of the superposition scenario at 20 Hz and short SOA, the positive components elicited by two consecutive stimuli overlapped resulting in an average waveform wider than expected based on other stimulation frequencies and SOAs (see superposition scenario in Fig. 9D). Similarly, in some particular cases (e.g. the 8.3 rev/s average waveform in Fig. 10A) the peak-to-peak latency seems to be overestimated. However, this apparent overestimation is rather due to the automatic procedure employed to detect local maxima/minima. In the particular case of the 8.3 rev/s waveform, although there was a deflection at approximately 70 ms from stimulus onset (most likely corresponding to the N75 component), this deflection did not fulfil the criteria for local minima.

Taken together, this pattern of results showing a stable transient ERP shape, seems more compatible with the above superposition scenario than with the existence of local entrainment in the jitter sequences (see Fig. 9D, 10C). This was confirmed by a 2-way ANOVA, revealing no main effects of either global stimulation frequency (F_8,3_ = 1.527, p = .398) or preceding SOA (F_2,9_ = 0.069, p = .933).

#### Absence of additional responses beyond the last stimulus of the train

**Figure 11:**
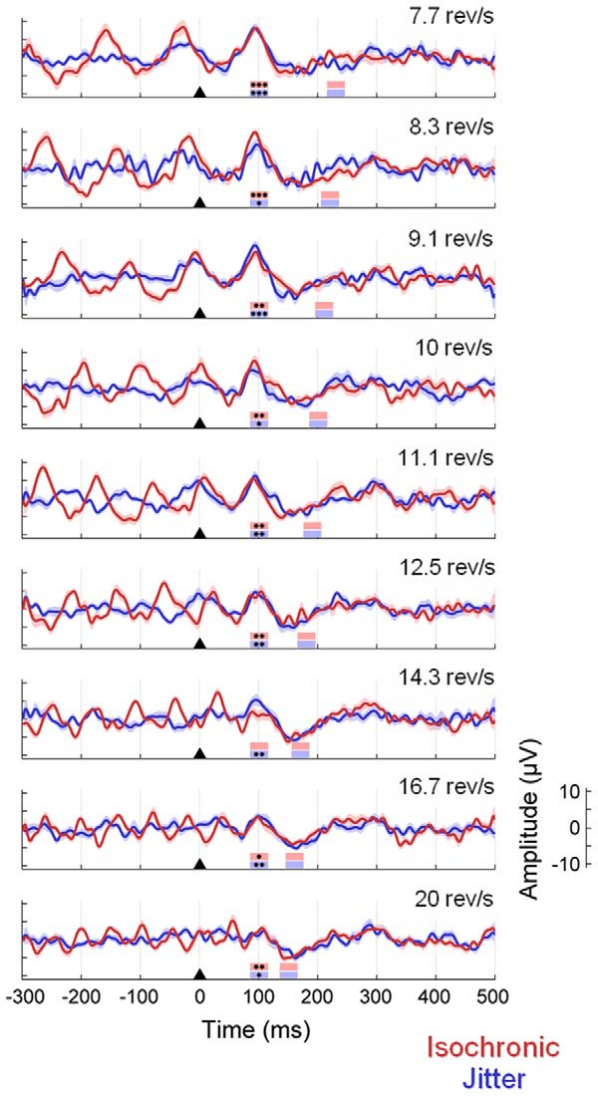
Absence of additional activity beyond the last stimulus of the sequence. The figure shows the grand-average ERPs time-locked to the last stimulus of the train for both isochronic (red lines) and jitter (blue lines) conditions from Experiment 2. The shaded area represents the standard error of the mean across subjects. Triangles indicate the onset of the last stimulus. The time window corresponding to the P100 component has been marked by the first column of coloured boxes (from 85 to 110 ms after stimulus onset; red: isochronic conditions; blue: jitter conditions). The time window corresponding to a potential additional response has been marked by the second column of coloured boxes, with different latency depending on stimulation rate. The asterisks indicate average responses significantly higher than 0 (*p<.05; **p<.01; ***p<.001); whereas the absence of asterisks indicates no significant responses (p>.05).

The grand-average ERPs time-locked to the last stimulus of the train at different stimulation rates are shown in Figure 11. As the figure shows, the expected response to the last stimulus of the sequence is significantly higher than 0 in most of the conditions. In contrast, there was no evidence of additional positive deflections at the stimulation rate beyond the end of the train. This result is compatible with the absence of oscillatory entrainment at the stimulation frequency.

## Discussion

The present study shows that visual steady-state responses (SSRs) can be accurately predicted from the linear summation of appropriately constructed transient responses. We demonstrate that the non-linear changes in neuronal responses can be fully accounted for by altered responses due to the stimulation history, which is represented in our transient responses obtained from jittered sequences. In two different experiments we replicate these findings for occipital SSRs and we show that they also hold for the whole scalp topographies elicited by steady-state stimulation. Furthermore, we demonstrate that our results are not better explained by an oscillatory entrainment mechanism. In summary, SSRs might simply correspond to a periodic succession of transient ERPs, indirectly suggesting that SSRs and ERPs are probably generated by the same underlying neural mechanism.

In the auditory modality, the nature of SSR generation has been a recurrent debate. This discussion was triggered by the study of Galambos and colleagues [11] who suggested that the auditory SSR could result from the linear summation of middle latency responses. Subsequent studies have shown contradictory results. While some studies have reported a high correspondence between recorded SSRs and those synthesized by linear addition of transient responses [15]–[17]; others have failed in accurately reconstructing SSRs from transients [18], [19]. Although these negative results seemed to rule out the superposition theory, the low correspondence between synthetic and recorded data has been proposed to be most likely due to a suboptimal template estimation procedure [15], [17]. The template traditionally used to reproduce SSRs is the average of transient responses at large SOAs that avoids interference between subsequent responses [2]. However, in a steady-state paradigm stimuli are not presented in isolation, but rather embedded in a stream of repeating stimuli. Consequently, if stimulus repetition had an effect on transient ERPs, the traditional template would not account for it and, hence, it would not provide an optimal estimate of the basic transient response underlying SSR generation. Indeed, repetitive stimulation produces non-linear changes in the amplitude and latency of the transient components [28]–[30]. Although the neural mechanisms responsible for these changes are unclear, two major explanations have been proposed: (i) refractoriness of the underlying neural generators [6], [19], [31], [32] and (ii) latent inhibition, implying that the transient excitation of the neural generators responding to the first stimulus in a sequence spreads to neurons that, in turn, feed back on them attenuating the response to incoming stimulus [33], [34]. It has been argued that the changes in the responses to individual stimuli with increasing stimulation rates provide evidence in favour of the non-linearity of the SSR and, consequently, against the linear mechanism postulated by the superposition theory [35]. However, as described above, these changes are rather caused by adaptation phenomena and, consequently, they are not exclusive to steady-state stimulation (i.e. high rate regular stimulation). For example, non-linear adaptation effects can also be observed when some jitter is introduced in the stimulation train [36] (see Fig. 4). Similarly, the mere repetition of a given stimulus at relatively low rates also produces a reduction in the amplitude of the transient response to the second stimulus of a pair (i.e. sensory-gating) [31], [37]. In addition, it is important to emphasize that the superposition hypothesis postulates that SSRs might result from the linear superposition of the transient responses elicited by individual stimuli; this does not at all imply that transient ERPs are invariant or unaffected by any non-linear mechanisms. In fact, an optimal transient template should take into account the non-linear effects caused by stimulus repetition to reflect the actual properties of the waveform at the stimulation rate of interest. As proposed by Bohorquez and colleagues [15], [16] optimal templates should be obtained by using jitter stimulation sequences close to steady-state rates. In the present study we tested two different strategies to construct jitter sequences. In Experiment 1, the amount of jitter was adjusted to each stimulation rate, with higher jitter for lower stimulation rates; whereas in Experiment 2, we employed a relatively low amount jitter that was set constant for all stimulation rates. Our results show that the latter approach is less efficient at obtaining optimal transient templates, as it failed to cancel out the activity elicited by preceding and forthcoming stimuli, especially at lower stimulation rates. Although this problem could be overcome by using a Gaussian modulation, the SSRs synthesized in Experiment 2 were slightly less accurate than those obtained from adjusted jitter sequences in the first experiment. To sum up, in the present study we have shown that the use of optimal transient templates obtained from the event-related response to adequate jitter sequences allows an effective reconstruction of SSRs from transient ERPs at a wide range of stimulation rates. Moreover, for the traditionally used template obtained from a low rate regular stimulation sequence, the SSR reconstruction is considerably less accurate (see Fig. S3), highly resembling the negative results previously reported [18], [19]. Taken together, our results therefore suggest that the assumed non-linearity between transient and steady-state responses [1], [3], [6] may rather be at the level of the non-linear adaptation phenomena that influence transient responses. When these non-linear effects are taken away, the relationship between transient ERPs and SSRs becomes linear, suggesting that they might not constitute two qualitatively different types of brain response.

The traditional understanding of SSR generation, which is in conflict with the superposition hypothesis, states that SSR results from a non-linear phase reorganization mechanism or the entrainment of an intrinsic neural rhythm that is more optimally driven at specific stimulation rates, the so-called resonance frequencies [8]–[10]. This hypothesis requires the following concepts that we will discuss in more detail below: (i) the phase reorganization mechanism, (ii) the driving phenomenon or entrainment of a neural rhythm, and (iii) the resonance phenomenon.

The mechanisms underlying the generation of event-related responses is still an open debate [38]–[40]. On one hand, the classic additive model states that ERPs comprise fixed latency and fixed polarity evoked responses that are additive to and independent of the background oscillatory brain activity [25], [41]–[44]. On the other hand, the oscillatory model highlights the role of phase resetting of ongoing oscillatory activity in the generation of ERPs [45]–[48]. Similarly, physiologically-based modeling has demonstrated that ERPs and ongoing EEG activity can be integrated within the same framework [49], [50]. It is important to emphasize that this study was not aimed to investigate the contribution of evoked and phase-resetting mechanisms in the generation of ERPs and SSRs, but rather to investigate the nature of the relationship between ERPs and SSRs. Consequently, the approach employed here has not been based on dissociating the role of evoked and phase-resetting mechanisms according to a set of agreed criteria [38], [44], [51], [52]. Nevertheless, although this study does not directly address the evoked/phase-resetting question, it might indirectly shed some light on this topic. While previous studies have investigated these generative mechanisms separately for ERPs and SSRs, the superposition theory might provide a unified framework to integrate both lines of research. As previously mentioned, the main claim of the superposition theory is that SSRs consist of sequentially overlapped ERPs. A prediction derived from this equivalence between steady-state and transient brain responses is that their generative neural mechanisms, whatever they are, should be the same. Indeed, previous studies seem to support this theoretical prediction. For instance, it has been demonstrated that the phase-resetting of ongoing oscillations plays a crucial role in the generation of both SSRs [53], [54] and transient ERPs elicited by repetitive stimulation [55].

The driving phenomenon refers to the entrainment of a neural rhythm at the same frequency as the driving stimulus train. The assumption that the brain is entrained at the stimulation frequency comes from the observation that SSRs are characterized by quasi-sinusoidal waveforms whose frequency spectra show a prominent peak at the fundamental frequency of stimulation. However, as we have shown in this study, synthetic SSRs generated from the linear superposition of transient responses occurring periodically show the same waveform and spectral pattern that characterize the driving phenomenon. This in turn implies that it is not possible to infer from a waveform or a spectrum whether a neural oscillation has been entrained at the stimulation rate or whether the brain has responded with a series of transient responses at that rate. However, this differentiation is essential for our understanding of brain functioning. The first alternative implies that an external sensory input can modify the oscillatory behavior of neuronal populations. In other words, even though there is a preferred or resonance frequency of a given neural network, this can be modified to a wide range of frequencies [10]. In contrast, the alternative explanation derived from the superposition hypothesis suggests that a given network always responds in a similar fashion (i.e. a transient response) and it is the rhythmicity of the stimulation that leads to the oscillatory components in the signal. Indeed, it has been recently demonstrated by means of transcranial magnetic stimulation that different cortical regions tend to be tuned to their characteristic or natural frequencies [56]. In summary, the superposition hypothesis suggests that the spectral pattern of the SSR does not necessarily result from the entrainment of a neural oscillation at the frequency of the sensory input. This suggestion is supported further by the absence of oscillatory entrainment at the stimulation frequency found in this study. Our results showed no evidence of immediate entrainment at the local stimulation rate, as well as no indication of longer-lasting neural activity beyond the stimulus train. However, it is important to notice that the absence of oscillatory entrainment at the stimulation frequency does not at all compromise the critical role of ongoing oscillatory activity in the generation of ERPs/SSRs, as it was discussed in the paragraph above.

Finally, the resonance phenomenon refers to the preference of a system to respond strongly to certain frequencies. The typical resonance frequencies of the visual system are 9–10, 16–18 and 40–50 Hz [2], [3], [10]. Each of them defines a so-called frequency subsystem (low, medium and high respectively) characterized by specific properties, latency and brain topology [57]. However, resonance-like peaks might appear as a consequence of phase interference phenomena between consecutive transient responses. If the stimulation rate matches up with the characteristic frequency of a given component of the transient response, this component will be preserved in the resulting waveform; in the opposite case, the component will be cancelled out. This mechanism has already been proposed to explain the 40 and 80–90 Hz resonance frequencies of the auditory SSR [11]. This explanation can also be applied to the visual system, given that its distinctive resonance frequencies are in principle similar to the constituent frequencies of the transient response. For instance, the components of the flash transient response are sequentially characterized by high (40–60 Hz), medium (14–20 Hz) and low (9–12 Hz) frequencies [57], resembling the three frequency subsystems of resonance. However, this is an open question given that the time-frequency pattern of the visual transient response at different repetition rates has not been characterized yet. More studies are needed to investigate whether the typical resonance frequencies for different stimulation types and sensory modalities correspond to the frequency characteristics of the corresponding transient responses, as it has been precisely demonstrated for the auditory SSR in a recent study [58]. In addition, future studies should investigate neural responses (in terms of phase and amplitude changes) to steady-state stimulation at the characteristic frequency of specific sensory areas.

This study provides evidence that visual SSRs can be precisely reconstructed by linearly adding the visual transient response elicited by every single stimulus in a stimulation train. This conclusion applies to a wide range of stimulation rates and parameters used, i.e. widely used square wave-modulated high contrast pattern reversal. The linear relationship between steady-state and transients suggests that they do not constitute two different modes of brain response. Furthermore, this explanation challenges the traditional understanding of the steady-state phenomenon as the ability of the brain to “follow” a stimulus or the stimulus to “drive” a brain response [1]. In summary, we conclude that the superposition of transient responses is a plausible and parsimonious mechanism underlying the genesis of SSRs.

## Supporting Information

Figure S1 Two representative subjects at two different stimulation rates (Experiment 1). The figure shows the transient template, the synthetic waveform and the recorded waveform for two subjects in the 7.1 rev/s and 20 rev/s conditions of Experiment 1.
(0.55 MB TIF)

Figure S2 Voltage topography for both recorded and synthetic data synchronized to 100 ms (Experiment 2). As figure 8, the figure shows the scalp voltage topographies for the dominant frequency of each condition (rows) and phase angle (columns). In this figure, however, phase angles are referred to the common positive component corresponding to P100 (i.e. 0° phase angle corresponds to 100 ms). Recorded topographies are shown in solid boxes; synthetic topographies are presented in the dashed boxes.
(1.55 MB TIF)

Figure S3 Synthetic data using the traditional transient template (Experiment 1). A, Traditional template for the transient response. The template was extracted from the isochronic condition with the largest SOA (2.7 rev/s isochronic condition). To remove the influence of subsequent responses (shaded in dark grey), the template comprised a 500 ms time window including 150 ms pre-stimulus activity (shaded in light grey). B, Grand-average waveforms synthesized from the traditional template (black line) in comparison to the recorded waveforms (grey line). Note that although the waveforms show similarities, the amplitude of the synthetic waveform is overestimated as the stimulation rate increases.
(0.38 MB TIF)
