# Supplementary figures and images for: Steady-State Visual Evoked Potentials Can Be Explained by Temporal Superposition of Transient Event-Related Responses
Source: PLoS One. 2011 Jan 18;6(1):e14543. doi: 10.1371/journal.pone.0014543 (PMC3022588; doi:10.1371/journal.pone.0014543)

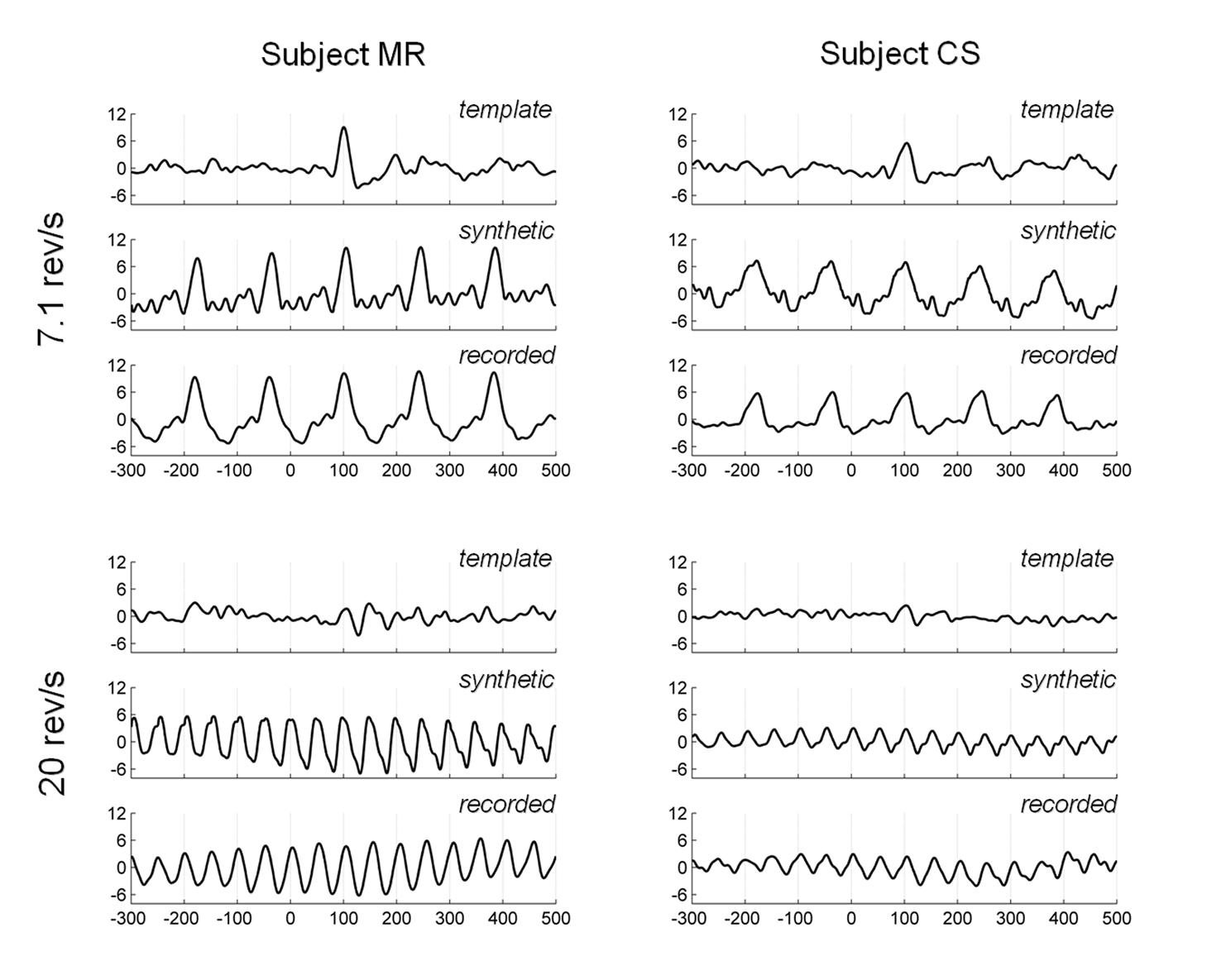

Supplement: Figure S1 — Two representative subjects at two different stimulation rates (Experiment 1). The figure shows the transient template, the synthetic waveform and the recorded waveform for two subjects in the 7.1 rev/s and 20 rev/s conditions of Experiment 1. (0.55 MB TIF) [file pone.0014543.s001.tif]

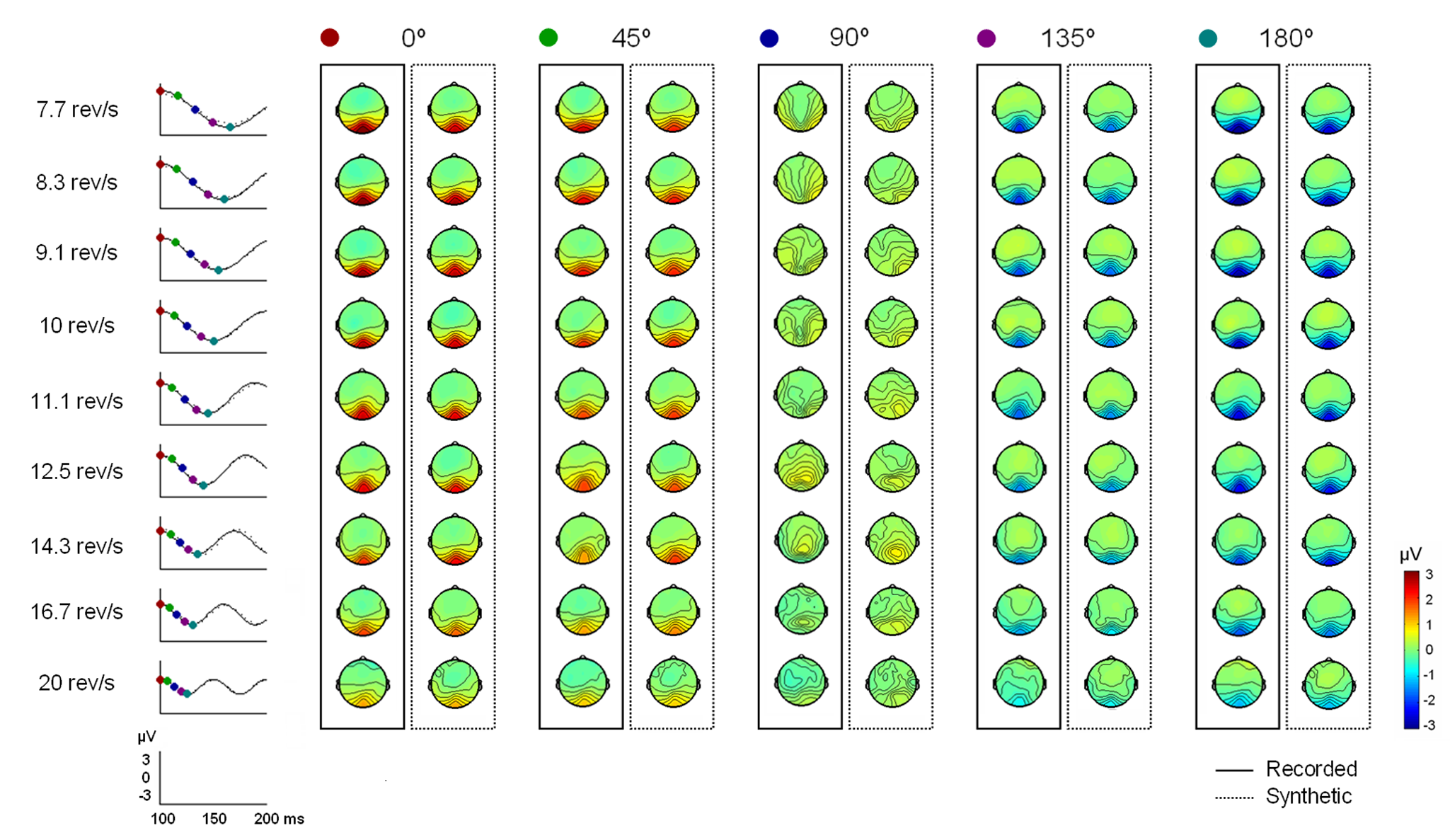

Supplement: Figure S2 — Voltage topography for both recorded and synthetic data synchronized to 100 ms (Experiment 2). As figure 8, the figure shows the scalp voltage topographies for the dominant frequency of each condition (rows) and phase angle (columns). In this figure, however, phase angles are referred to the common positive component corresponding to P100 (i.e. 0° phase angle corresponds to 100 ms). Recorded topographies are shown in solid boxes; synthetic topographies are presented in the dashed boxes. (1.55 MB TIF) [file pone.0014543.s002.tif]

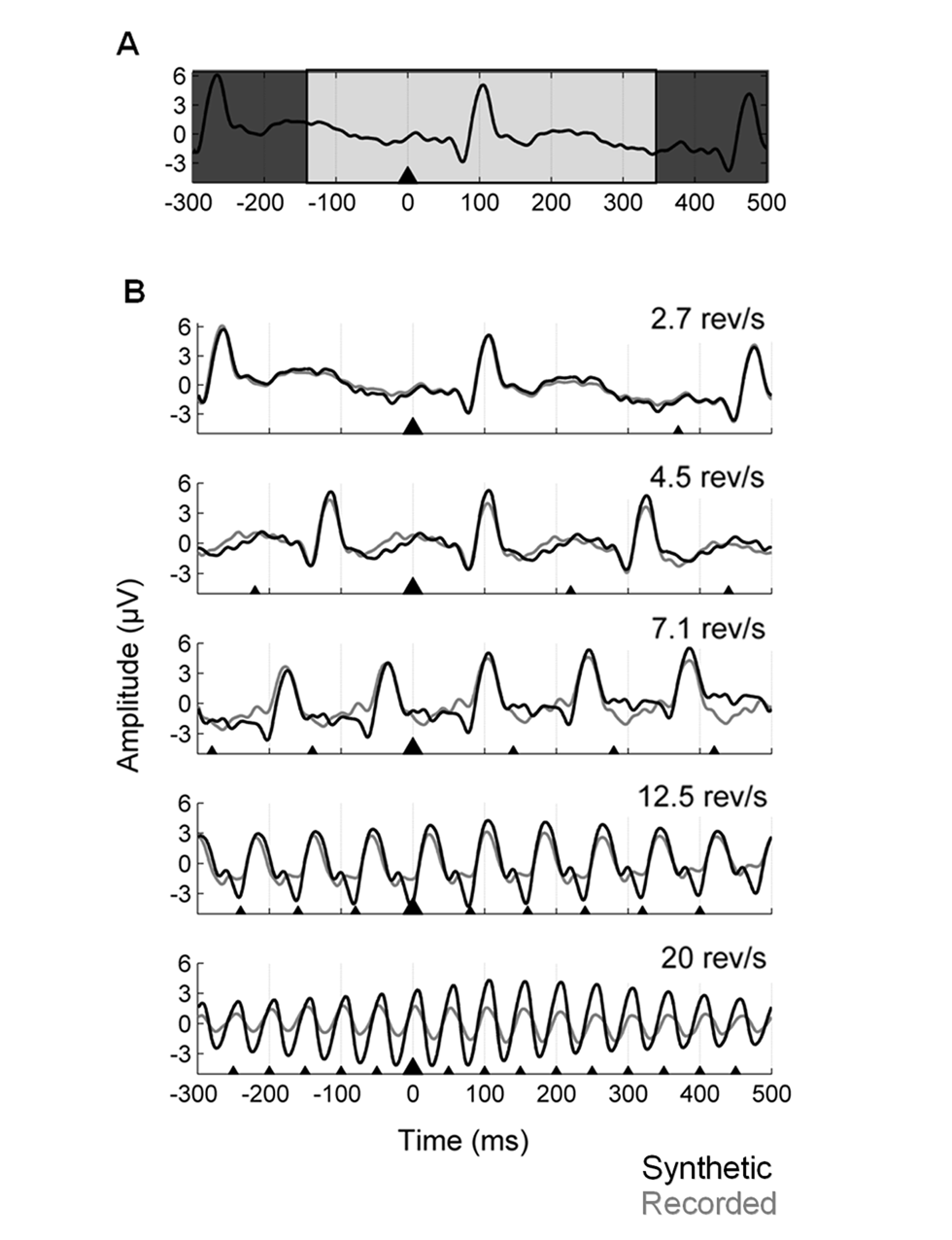

Supplement: Figure S3 — Synthetic data using the traditional transient template (Experiment 1). A, Traditional template for the transient response. The template was extracted from the isochronic condition with the largest SOA (2.7 rev/s isochronic condition). To remove the influence of subsequent responses (shaded in dark grey), the template comprised a 500 ms time window including 150 ms pre-stimulus activity (shaded in light grey). B, Grand-average waveforms synthesized from the traditional template (black line) in comparison to the recorded waveforms (grey line). Note that although the waveforms show similarities, the amplitude of the synthetic waveform is overestimated as the stimulation rate increases. (0.38 MB TIF) [file pone.0014543.s003.tif]
